# Multiple Micronutrient Supplementation: As a Supportive Therapy in the Treatment of COVID-19

**DOI:** 10.1155/2022/3323825

**Published:** 2022-03-14

**Authors:** A Salomy Monica Diyya, Noel Vinay Thomas

**Affiliations:** ^1^College of Medicine, Department of Pharmacy, Komar University of Science and Technology, Sulaymaniyah, Kurdistan, Iraq; ^2^College of Science, Department of Biomedical Science, Komar University of Science and Technology, Sulaymaniyah, Kurdistan, Iraq

## Abstract

During the infection and treatment of the SARS-CoV-2 viral infection, age and comorbidities play a major role in the successful management of COVID-19. The nutritional status changes which occur in the body vary with the age and underlying conditions and has a vital role in the functioning of the immune system and cellular membrane integrity, thus minimizing the vulnerability to the infection. Considering the data already published by eminent researchers, a few micronutrients have shown outstanding results as supportive therapies in the treatment of viral infections. Micronutrient like zinc improves the membrane barrier integrity, has anti-inflammatory activity, and is involved in antibody production. Vitamin A supports the phagocytic activity of macrophages, while vitamin C reduces the worsening of respiratory tract infections by restoring the dysfunctional epithelial barrier of the lungs. Vitamin D, vitamin E, selenium, and omega-3 fatty acid metabolites play a major role in immunomodulation and in the inhibition of proinflammatory cytokine production. Magnesium is involved in the synthesis of antibodies, while copper, vitamin B12, and folate have significant effects on immune cells. A few researchers suggest that iron supplementation has reduced the risk of acquiring respiratory tract infections in children. As the age of the patient increases, the need for micronutrients increases, thus leading to an imbalanced nutritional status which in turn increases the risk and fatality of the infections. The use of micronutrients in modulating the inflammatory, immune responses, and the epithelial barrier integrity is explored during the treatment of viral infections for faster recovery.

## 1. Introduction

The coronavirus, severe acute respiratory syndrome coronavirus type 2 (SARS-CoV-2), has appeared in Wuhan, China, for the first time in late 2019, and the people who are infected with the SARS-CoV-2 virus suffer from severe respiratory illness.

Indeed, the elderly and people with comorbidities are the most vulnerable to COVID-19 [1]. With age and age-related nutritional status, there will be changes in the integrity of the membrane (physical barrier) and these will disrupt the immunological responses. These changes may suppress the immune function and increase the chances of getting infected [2]. The vitamins and minerals neutralize the damage caused by oxidative agents, thus helping us to understand the relationship between nutrition and infection rate [3]. The immunosuppression and tight junction impairment are seen in the initial stage of COVID-19 infection, which is evidenced by the changes at the molecular level [4]. The major reasons for the hypoxemia in acute respiratory distress syndrome (ARDS) are alveolar edema and the formation of exudate because of increased permeability of the alveolar epithelial barrier [5]. The permeability of the lung epithelium depends on a set of tight junctions' heteromeric complexes, which help in sealing the interface between adjacent epithelial cells [4]. Tight junctions are also reported between the epithelial cells in organs like the kidney, brain, and intestine [6, 7]. During virus infection, the breakdown of the epithelial barrier is seen as a result of the disruption of tight junction complexes [8]. Certain micronutrients have an ability to build a less leaky tight junction complex by changing the composition and structure of the tight junction, thus enhancing the epithelial barrier integrity [9].

Micronutrients, along with their role in the maintenance and development of physical barriers, assist in enhancing immune function by being involved in the production of antimicrobial proteins and mediation of inflammatory processes [10]. Vitamin and mineral supplements help in neutralizing the damaging effects of a few oxidative agents that can damage cells [11]. Deficiencies of certain nutrients suppress the immune function and increase the susceptibility to various infections [12–14].

## 2. Role of Micronutrients in Modulating the Cell Membrane Barrier Integrity and Immune Response

### 2.1. Zinc

Zinc supplementation has improved barrier integrity along with specific changes in tight junction complexes. Thus, zinc supplementation can also alleviate the proinflammatory effect of cytokines, thus preventing the impairment of barrier integrity. [15–17]. Zinc plays a major role in modulating innate and adaptive immunity responses and is also involved in cytokine production [18]. Thus, high levels of zinc are associated with a vulnerability to infection [19]. A few researchers proved that zinc is also involved in the production of IgG antibodies [20].

Zinc has both pro- and anti-inflammatory effects. An optimum intake of it helps in limiting the overproduction of inflammatory cytokines. Zinc supplementation is found to have a direct antiviral effect on respiratory syncytial viruses, dengue viruses, and coronaviruses [21]. Chloroquine's toxicity on viruses is enhanced when chloroquine is administered with zinc [22]. A few researchers reported that combining Zn^2+^ and zinc-ionophores (such as pyrithione) can increase intracellular zinc ion concentration, which aids in SARS-coronavirus inhibition [21]. As a result, zinc supplementation in combination with antiviral or antimicrobial agents has the potential to be beneficial [23].

### 2.2. Vitamin A

The innate immune response and cell-mediated immunity are regulated by Vitamin A. It is also involved in cytokine signaling and in humoral antibody immunity [22]. Compromised mucosal epithelium integrity is observed in vitamin A deficiency that leads to increased susceptibility to infection via the eyes, respiratory, and gastrointestinal tract [24]. Thus, vitamin A deficiency leads to increased susceptibility to infections. Vitamin A has a role in the antimicrobial action of macrophages with the oxidative burst and phagocytic activity [25]. Vitamin A supports normal growth and differentiation of B cells [26]. Antibody production is augmented by the effect of vitamin A on the development of T helper 2 cells and antigen-presenting cells [27, 28]. Retinoic acid, a metabolite of Vitamin A, helps in the activation of B cells that are produced by the gut, thus influencing the proper functioning of B cells. The activated B cells express high levels of a factor, a gut-homing receptor, which helps in maintaining the balance between immunity and intestinal tolerance. Scientific evidence shows that Vitamin A is necessary for IgA antibody response towards bacterial antigens via B cell-mediation [29].

### 2.3. Vitamin C

As an antioxidant, vitamin C improves the activity and function of immune cells, the migration of white blood cells, and the function of leucocytes [30]. Thus, vitamin C deficiency impairs immunity, increases susceptibility to contracting pneumonia, and worsens disease severity [31]. A little research evidence show the restoration of the dysfunctional epithelial barrier of the lungs in animals is observed upon vitamin C administration [32]. Thus, supplementation with vitamin C can minimize the risk of acquiring respiratory tract infections. Vitamin C, being a water-soluble antioxidant, plays a vital role in balancing the cellular redox homeostasis, thus protecting the host cells against the detrimental effects of reactive oxygen species [12] that are released by the phagocytes and ultimately lead to the deactivation of viruses and bacterial death. Vitamin C also modulates the proinflammatory signaling pathway that is activated by the oxidative burst [33] and influences innate immunity by regulating neutrophil function [34]. Vitamin C influences neutrophil apoptotic processes in the inflammation pathway, thus reducing the extent of tissue damage [35]. Ascorbic acid may lessen tissue damage by reducing the formation of neutrophil extracellular trap nets [36]. Followed by SARS-CoV-2 infection, the dysfunction of T cells is observed [37]. Linked to this, the latest research explains the role of vitamin C in T-cell maturation through epigenetic mechanisms [38]. A few in vitro studies explain the role of ascorbic acid as a potent immune stimulator in antibody production like IgM and IgG, thus supporting the fact that the intracellular ascorbic acid concentration plays a major role in establishing the immune response of the peripheral blood lymphocytes [39]. As a result, vitamin C appears to play a role in viral clearance via T cells.

### 2.4. Vitamin D

Vitamin D helps in the production of antimicrobial peptides and thus takes part in innate immune responses [40]. The lower the levels of vitamin D, the higher the levels of proinflammatory cytokines [41]. Thus, these results indicate the association of vitamin D with inflammatory process activation. Vitamin D induces the production of antimicrobial peptides by the cells of the immune system and respiratory epithelium [42]. It inhibits the production of proinflammatory cytokines and hence can be linked to COVID-19 treatment. The pathogenicity of respiratory viruses like SARS-CoV-2 causes a condition called cytokine storm [43]. Thus, the immunomodulatory effect of vitamin D plays a major role in the treatment of viral infection. Supporting this, a recent study has shown a noticeable reduction in intensive care admissions of COVID-19 patients upon administration of high doses of 25-hydroxyvitamin D [44]. Vitamin D upregulates mitogen-activated protein kinase (MAPK) phosphatase 1 (MKP-1) which decreases the macrophagic cytokines. On the other hand, vitamin D is involved in alleviating the hyperinflammation induced by tumor necrosis factor-alfa (TNF-alfa) by decreasing the production of MMP-9 (matrix metalloproteinase-9) [45].

### 2.5. Vitamin E

The protection of cell membrane integrity from detrimental effects of free radicals is observed upon vitamin E supplementation [46]. Supplementing with vitamin E also improves innate natural killer (NK-cell) cell response [47].

### 2.6. Selenium

Selenium acts as a cellular antioxidant and is involved in the immune response by acting on selenoproteins. It also has a major role in modulating cytokine production and inflammatory responses. Selenium supplementation increases the immune system response towards viruses [48], and this is evidenced by the relationship between selenium deficit levels and increased susceptibility to viral infections [49].

## 3. Role of Micronutrients in Minimizing the Incidence of Acute Infections

An indirect relationship is drawn by a research group between immune function and risk of infection by considering a study that includes selenium deficit mice. In which the Coxsackie B3 virus mutates into a more virulent strain that results in severe cardiomyopathy [50]. They also observed that the damage caused by the influenza virus is more severe in the lungs of selenium-deficient mice. These studies suggest that low levels of selenium affect the severity of viral infection. As a result, these findings pave the way for further research into the effect of selenium on viral virulence in humans. Thus, the deficiency of certain micronutrients leads to increased susceptibility to infections and the fatality of certain infections. A few individuals who have certain disease conditions like inflammatory bowel disease, chronic inflammation, and malabsorptive conditions are more prone to micronutrient deficiency [51]. In these ill conditions, an increased need for micronutrients is observed, which can be overcome by their supplementation. Elderly individuals are more prone to infections as a phenomenon of immunosenescence is observed in which a low response to vaccination is seen because of low production of IgA and diminished immune cell activity [52, 53]. Hence, micronutrient supplementation in elderly individuals helps in overcoming the severity of infections, as elderly people are at higher risk of micronutrient deficiencies. This is evidenced in a few hospitalized elderly individuals. Upon vitamin C supplementation, a decreased severity of pneumonia is observed [54] and enhanced immune responses are evidenced with vitamin E supplementation [54, 55]. Elevated levels of vitamin D are linked with lower levels of proinflammatory cytokines. Thus, vitamin D is associated with the mediation of inflammation. Vitamin D activation in the lung can attenuate the inflammatory cytokines in response to viruses during lung infections [41]. Upon supplementation, vitamin A noticeably reduced the incidence of lower respiratory tract infections [56]. In children and adults, vitamin D supplementation reduced the risk of upper respiratory tract infections, influenza, and tuberculosis in children and adults [57]. The impaired immune system functioning and increased susceptibility to infection can be treated by compensating for the deficit levels of micronutrients through supplementation. Thus, micronutrients play a major role in the treatment of COVID-19 as they support many stages of immune response [58].

### 3.1. Omega-3 Fatty Acids

Resolvins, the metabolites of omega-3 fatty acids, are effective in inhibiting the migration of neutrophils, thus reducing the entry of neutrophils into the inflammation site [59]. Specialized proresolving mediators (spm) from omega fatty acids trigger granulocyte apoptosis by stimulating natural killer cells [59]. The Spm also promotes the anti-inflammatory response by suppressing cytokine production.

### 3.2. Copper

Copper can destroy a wide range of microorganisms by exhibiting intrinsic antimicrobial properties [60]. Complement proteins enhance the opsonization of pathogens by phagocytes. And the serum levels of complement proteins are enhanced by vitamin C [61]. Micronutrients like vitamin A, C, E, vitamin B6, B12, zinc, and folate maintain and enhance the cytotoxic activity of NK cells, which play a major role in attacking host cells that exhibit abnormal plasma membrane proteins [62].

### 3.3. Magnesium

Magnesium acts as a cofactor in the synthesis of antibodies [63]. Meanwhile, magnesium reduced the production of superoxide anions by regulating the activation of peripheral blood neutrophils and eosinophils in eosinophilic patients [64].

Vitamin B12 is needed for cell division and replication. As a result, its role in the rapid proliferation of B cells is explained.

### 3.4. Folic Acid

Folic acid levels must be adequate to generate an appropriate antibody response to antigen [65].Thus, multiple micronutrient supplementations have a significant effect on immune cells and responses [66].

### 3.5. Iron

**Figure 1:**
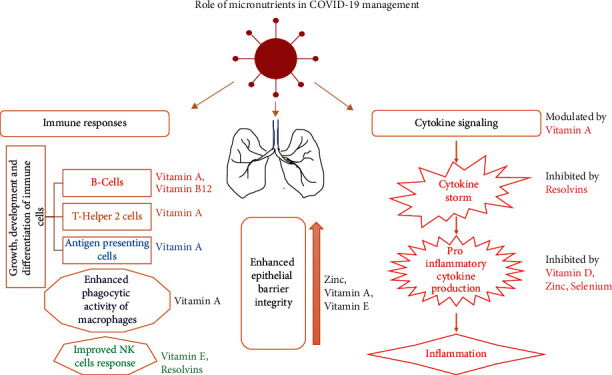
Role of micronutrients in COVID-19 management.

Iron supplementation in children reduces the risk of respiratory tract infections [67]. Because the severely affected COVID-19 patients experienced hyperinflammation and cytokine storm [68], micronutrient supplementation could be beneficial in COVID-19 management. The various effects of micronutrients in the immune and inflammation regulatory pathways are explained in Figure 1, and the recommended dose of nutrients is given in Table 1.

## 4. Conclusion

Considering the fact that elderly people and people with comorbidities are more prone to SARS-CoV-2 infection, the role of micronutrients is explored in alleviating the symptoms and improving the immune system of the body. The nutritional status of the body changes as age progresses, which in turn disrupts the immune responses, thus paving the way for the need for micronutrient supplementation. Micronutrients play a significant role in maintaining the cellular membrane integrity and permeability, especially in the alveolar epithelial barrier of the lungs. During any viral infection, disruption of tight junctions of the epithelial barrier is observed. Hence, micronutrients are considered important as they help in building the integrity of the epithelial barrier. They are helpful in the production of antimicrobial proteins and are also involved in inflammatory processes. Micronutrients neutralize the damaging effects of oxidative agents on cells. Every stage of the immune response relies on the availability of certain micronutrients. Certain micronutrients like vitamins A, C, D, E, and zinc are required to ensure the structural and functional integrity of the skin and mucus membranes as they form the first line of defense as physicochemical barriers against invading pathogenic microorganisms. Cell-mediated innate immunity, complement activation, and the release of proinflammatory cytokines are all heavily reliant on adequate levels of vitamins A, B12, C, D, E, folic acid, and minerals such as iron, zinc, selenium, magnesium, and copper. According to some research, low selenium levels increase susceptibility to viral infection. Parallelly, certain micronutrients like vitamin E, C, zinc, magnesium, iron, selenium, and copper are involved in the self-protection of immune cells by certain antioxidant mechanisms. Copper has intrinsic antimicrobial properties. While magnesium acts as a cofactor in antibody synthesis, Optimum levels of vitamin B12 and folate are required for proper antibody responses toward antigens. The metabolites of omega-3 fatty acids, resolvins, show an anti-inflammatory response and are involved in the stimulation of natural killer cells. Hence, a balanced immune system which is well maintained by certain specific micronutrients helps in reducing the risk of acquiring infections related to respiratory tract and viral infections and also helps in recovery at the molecular level when given as supplements along with antiviral medication. Thus, the deficiency of these micronutrients imparted a greater risk during the outbreak of SARS-CoV-2. The challenges faced during COVID-19 treatment can be overcome by micronutrient supplementation, keeping in consideration their increased demands of need with respect to age and various ill health conditions. By considering the data from observational and in vitro experimental studies conducted by renowned researchers, the role of micronutrient supplementation as supportive therapy along with antiviral or antimicrobial medication can be strongly established for safe and faster recovery.

## Figures and Tables

**Table 1:** Daily recommended dose of nutrients.

Nutrient	Absorbable form (oral administration)	Daily recommended dose intake (men)	Daily recommended dose intake (women)	Dietary sources
Zinc	Zinc picolinate, zinc citrate, zinc acetate, zinc glycerate, and zinc gluconate	11 mg	8 mg	Oysters, red meat, poultry, fortified breakfast cereals, beans, nuts, whole grains, and dairy products.
Vitamin A	Preformed vitamin A (retinol and its esterified form), provitamin A carotenoids	900 mcg	700 mcg	Beef liver, organ meats, fish, green leafy vegetables, fruits like cantaloupe, apricots and mangoes, dairy products, and fortified breakfast cereals.
Vitamin C	Mineral ascorbates like calcium ascorbate, zinc ascorbate.	90 mg	75 mg	Citrus fruits, tomatoes, sweet potato, broccoli, cabbage, and dark green leafy vegetables.
Vitamin D	Alfacalcidol, calcifediol, calcitriol, and dihydrotachysterol	15 mcg	15 mcg	Fortified milk, fortified breakfast cereals, fatty fish, fish liver oil, beef liver, egg yolks, and cheese.
Vitamin E	Alpha-tocopherol	15 mg	15 mg	Peanuts, almonds, olive oil, meats, dairy, leafy greens, and fortified cereals.
Selenium	Dietary selenium, selenomethionine	40-70 mcg	45-55 mcg	Milk, yogurt, fortified cereals, pork, beef, Turkey, shellfish, chicken, and eggs.
Omega-3 fatty acids	Reesterified triglyceride form	1600 mg	1100 mg	Cold water fatty fish, nuts, seeds, plant and oils.
Copper	Chelated or citrated copper	900 mcg	900 mcg	Oysters, shellfish, organ meats, dark leafy greens, dried fruits like prunes, cocoa, and black pepper.
Magnesium	Magnesium chloride	400-420 mg	310-320 mg	Greens, nuts, seeds, dry beans, whole grains, wheat germ, wheat, and oat bran.
Vitamin B12	Methylcobalmin	2.4 mcg	2.4 mcg	Meat, fish, milk, cheese, eggs, and fortified breakfast cereals.
Folic acid	Folic acid	400 mcg	400 mcg	Beef liver, asparagus, brussels sprouts, dark green leafy vegetables like spinach, mustard greens, oranges, nuts, beans, and peas.
Iron	Ferrous sulphate	8 mg	18 mg	Lean meat, seafood, poultry, lentils, spinach, kidney beans, nuts, dried fruits like raisins, iron-fortified breakfast cereals, and breads.

## Data Availability

The data that support the findings of this study are available from the corresponding author upon reasonable request.
